# *EGFR* Mutations in Surgically Resected Fresh Specimens from 697 Consecutive Chinese Patients with Non-Small Cell Lung Cancer and Their Relationships with Clinical Features

**DOI:** 10.3390/ijms141224549

**Published:** 2013-12-17

**Authors:** Yuanyang Lai, Zhipei Zhang, Jianzhong Li, Dong Sun, Yong’an Zhou, Tao Jiang, Yong Han, Lijun Huang, Yifang Zhu, Xiaofei Li, Xiaolong Yan

**Affiliations:** 1 Department of Thoracic Surgery, Tangdu Hospital, Fourth Military Medical University, Xi’an 710038, Shaanxi, China; E-Mails: lai_yuanyang@163.com (Y.L.); zzpchest@163.com (Z.Z.); zhou.yongan@163.com (Y.Z.); jted@soho.com (T.J.); han-yong@live.cn (Y.H.); hljyxq@fmmu.edu.cn (L.H.); wjtm1981@163.com (Y.Z.); 2 Department of Cardiothoracic Surgery, Binghua Hospital, Harbin 150080, Heilongjiang, China; E-Mail: jianzhong-0520@163.com; 3 The First Outpatient Department of Guangzhou Military Command of PLA, Guangzhou 510080, Guangdong, China; E-Mail: sdwsj@sina.com.cn

**Keywords:** *EGFR* mutations, NSCLC, targeted therapy, ARMS, surgery, fresh tumor specimens

## Abstract

We aimed to reveal the true status of epidermal growth factor receptor (*EGFR*) mutations in Chinese patients with non-small cell lung cancer (NSCLC) after lung resections. *EGFR* mutations of surgically resected fresh tumor samples from 697 Chinese NSCLC patients were analyzed by Amplification Refractory Mutation System (ARMS). Correlations between *EGFR* mutation hotspots and clinical features were also explored. Of the 697 NSCLC patients, 235 (33.7%) patients had tyrosine kinase inhibitor (TKIs) sensitive *EGFR* mutations in 41 (14.5%) of the 282 squamous carcinomas, 155 (52.9%) of the 293 adenocarcinomas, 34 (39.5%) of the 86 adenosquamous carcinomas, one (9.1%) of the 11 large-cell carcinomas, 2 (11.1%) of the 18 sarcomatoid carcinomas, and 2 (28.6%) of the 7 mucoepidermoid carcinomas. TKIs sensitive *EGFR* mutations were more frequently found in female patients (*p* < 0.001), non-smokers (*p* = 0.047) and adenocarcinomas (*p* < 0.001). The rates of exon 19 deletion mutation (19-del), exon 21 L858R point mutation (L858R), exon 21 L861Q point mutation (L861Q), exon 18 G719X point mutations (G719X, including G719C, G719S, G719A) were 43.4%, 48.1%, 1.7% and 6.8%, respectively. Exon 20 T790M point mutation (T790M) was detected in 3 squamous carcinomas and 3 adenocarcinomas and exon 20 insertion mutation (20-ins) was detected in 2 patients with adenocarcinoma. Our results show the rates of *EGFR* mutations are higher in all types of NSCLC in Chinese patients. 19-del and L858R are two of the more frequent mutations. *EGFR* mutation detection should be performed as a routine postoperative examination in Chinese NSCLC patients.

## 1. Introduction

Lung cancer remains the leading cause of cancer morbidity and mortality in males, comprising 17% of the total new cancer cases and 23% of the total cancer deaths. On the other hand, lung cancer is the fourth most commonly diagnosed cancer and the second leading cause of cancer death. In developing countries, the mortality burden for lung cancer accounts for 11% of the total female cancer deaths, as high as the burden for cervical cancer [1]. Surgery is the predominant approach for treatment of lung cancer, in combination with other approaches depending on the disease status. In recent years, targeted drug treatment has become a highlight for lung cancer, especially with the use of epidermal growth factor receptor-tyrosine kinase inhibitors (EGFR-TKIs).

Targeting EGFR is a promising strategy for the treatment of non-small cell lung cancer (NSCLC). Previous studies have demonstrated various *EGFR* mutations in NSCLC, including adenocarcinomas and nonadenocarcinomas [2–13]. Unlike drug resistant mutations (Exon 20 T790M point mutation (T790M) and exon 20 insertion mutation (20-ins)), NSCLC harboring EGFRTKI sensitive mutations such as exon 19 deletion mutation (19-del) and exon 21 L858R point mutation (L858R) respond to EGFR-TKIs [14]. Other drug sensitive mutations consist of exon 21 L861Q point mutation (L861Q) and exon 18 G719X point mutations (G719X, including G719C, G719S, G719A). Large-scale studies have also demonstrated that TKIs could apparently improve the therapeutic outcome of patients with *EGFR*-mutant NSCLC [12,15,16]. Thus, screening for *EGFR* mutations in NSCLC is significant in the decision-making on the treatment of NSCLC.

Surgically resected tumor specimens are the optimal DNA source for *EGFR* mutation detection. Complete and sufficient DNA can be extracted from surgically resected fresh samples, while specimens obtained from transbronchial lung biopsy or percutaneus aspiration lung biopsy could not demonstrate the whole tumor genomics because of the existence of intratumor genetic heterogeneity [17]. Surgical specimens can show relatively complete tumor genomics, thus avoiding or reducing false-negative results of *EGFR* mutation detections.

The amplification refractory mutation system (ARMS) is a method for point mutation in DNA based on allele specific polymerase chain reaction [18]. It is quick, relatively easy and more sensitive than DNA direct sequencing, and *EGFR* mutation detection kits according to the principle of ARMS can detect 29 common *EGFR* mutations [19–21]. As some mutations may be present in a minor population of tumor cells, and normal cells can be mixed in tumor tissue, this highly sensitive assay can be an appropriate method for *EGFR* mutation analysis.

The frequency of *EGFR* mutations in NSCLC is known to be associated with many factors, including race, gender, smoking status, and tumor histology. About 8%–15% of European patients with NSCLC harbor *EGFR* mutations. In Asian patients, the frequency is up to 31% and the mutations are associated with Asian ethnicity, female gender, never-smoking or light-smoking, and adenocarcinoma [7,12,13]. As most previous large-scale studies on *EGFR* mutations primarily focused on adenocarcinoma, few studies have evaluated the frequency of *EGFR* mutations in non-adenocarcinoma NSCLC, such as squamous-cell carcinoma, adenosquamous carcinoma, and large-cell carcinoma. In this study, we used ARMS to demonstrate the status of *EGFR* mutations in Chinese patients with NSCLC after lung resection and clarify correlations between *EGFR* mutations and clinical features.

## 2. Results

### 2.1. Patient Characteristics

Patient characteristics are summarized in Table 1. The 697 enrolled patients consisted of 476 males (68.3%) and 221 females (31.7%) with a median age of 55.3 years (range, 38–76 years), and 366 smokers (52.5%) *vs.* 331 non-smokers (47.5%). Postoperative pathological evaluation revealed 282 squamous-cell carcinomas (40.5%), 293 adenocarcinomas (42.0%), 86 adenosquamous carcinomas (12.3%), 11 large-cell carcinomas (1.6%), 18 sarcomatoid carcinomas (2.6%), and 7 mucoepidermoid carcinomas (1.0%). All the pathological diagnoses were based on the criteria of the WHO/IASLC Histological Classification of Lung and Pleural Tumors. According to the seventh edition of the TNM Classification of malignant tumors, cases in stage Ia, Ib, IIa, IIb, IIIa, IIIb, IV were 105, 110, 153, 159, 155, 12, respectively.

### 2.2. *EGFR* Mutation Status

**Figure 1.**
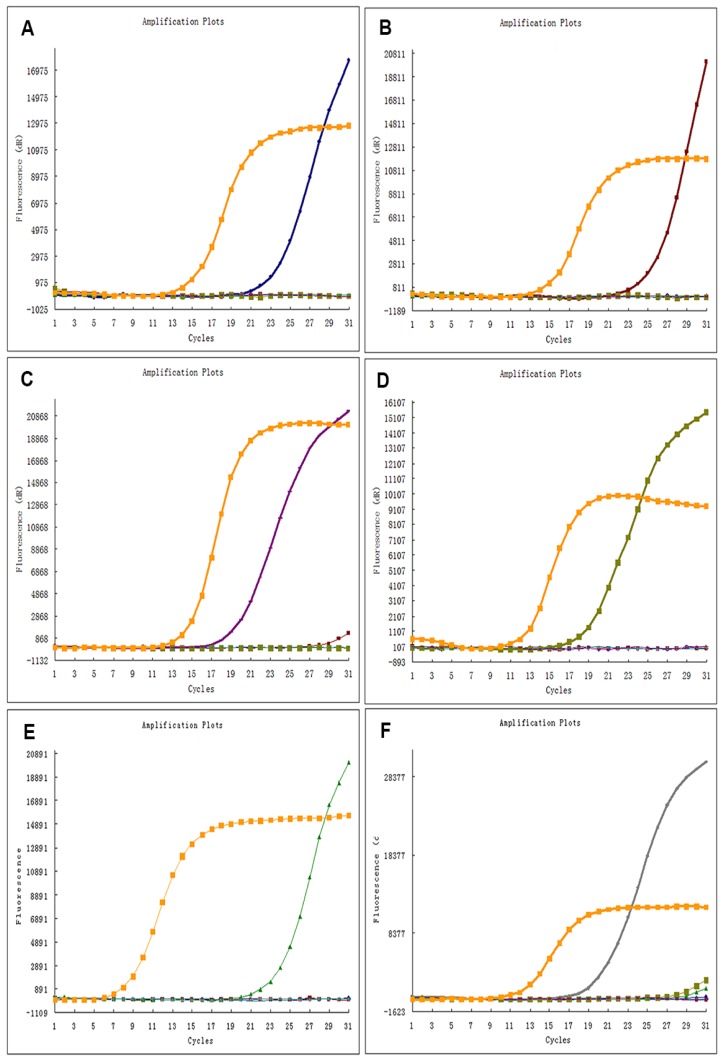
Representative results for ADx-ARMS of *EGFR* mutation testing. (**A**–**F**) demonstrate the typical results of exon 19 deletion mutation, exon 21 L858R point mutation, exon 21 L861R point mutation, exon 18 G719X point mutation, exon 20 T790M point mutation, exon 20 insertion mutation, respectively. The orange lines are the internal control signal (HEX), the blue lines, red lines, purple lines, dark green lines, light green lines and the gray lines are mutant signal (FAM), representing 19-del, L858R, G719X, T790M and 20-ins, respectively.

Typical results for ADx-ARMS are demonstrated in Figure 1. Of the 697 Chinese patients with NSCLC, 235 (33.7%) patients had TKIs sensitive *EGFR* mutations, comprising 41 (14.5%) of the 282 squamous-cell carcinomas, 155 (52.9%) of the 293 adenocarcinomas, 34 (39.5%) of the 86 adenosquamous carcinomas, 1 (9.1%) of the 11 large-cell carcinomas, 2 (11.1%) of the 18 sarcomatoid carcinomas, and 2 (28.5%) of the 7 mucoepidermoid carcinomas (Table 1). As shown in Tables 2–4, exon 19 deletion and exon 21 L858R point mutation were two of the most common subtypes of *EGFR* mutations, accounting for 43.4% (*n* = 102/235) and 48.1% (*n* = 113/235) respectively. L861Q was detected in 4 (1.7%) and G719X was detected in 16 (6.8%) of the 235 EGFR mutant patients. In addition, *EGFR* T790M mutation was identified in 6 patients and *EGFR* exon 20 insertion mutation in 2 patients. The data of the 8 patients are shown in Table 5. No coexisting mutations were found in our study.

Furthermore, a significant correlation was noticed between TKIs sensitive *EGFR* mutations and the clinical features of the female gender (*p* < 0.001), non-smoking history (*p* = 0.047) and adenocarcinoma subtype (*p* < 0.001), while patient age and tumor stage were not significantly associated with *EGFR* mutations (*p* = 0.060, *p* = 0.584 respectively). In addition, no significant difference was observed between the subtypes of TKIs sensitive *EGFR* mutations and gender (*p* = 0.634), smoking status (*p* = 0.349), and histology of tumors (*p* = 0.819) in a subgroup analysis where only mutation-positive patients were included (Tables 2–4).

## 3. Discussion

The present study was intended to improve our understanding about the *EGFR* mutation status in NSCLC, especially in non-adenocarcinoma.

Our results showed that the overall frequency of TKIs sensitive *EGFR* mutations was 33.7%, and the *EGFR* mutation rate in squamous-cell carcinoma (14.5%), adenocarcinoma (52.9%) and adenosquamous carcinoma (39.5%) was higher than the data from previous Asian population-based studies [2,6–9,13]. We also identified that one of the 11 large-cell carcinomas, 2 of the 18 sarcomatoid carcinomas and 2 of the 11 mucoepidermoid carcinomas harbored TKIs sensitive EGFR mutations. Meanwhile, we found that TKIs sensitive *EGFR* mutations were associated with the female gender (*p* < 0.001), non-smoking history (*p* = 0.045), and adenocarcinoma subtype (*p* < 0.001).

There are several underlying causes for the higher frequency of TKIs sensitive *EGFR* mutations in our study. Fresh and sufficient tumor samples after lung resection could be one of them. Owing to tumor heterogeneity and limitations of needle biopsy, the small amount of tissue from needle biopsies may not be emblematic of the complete pictures of tumors, leading to false negative results; while adequate surgical samples can provide enough DNA for *EGFR* mutation detection. On the other hand, as formaldehyde may cause crosslinking and degradation of DNA, surgically resected fresh samples can offer less damaged DNA than the formalin-fixed ones, thus making the results of *EGFR* mutation detection more accurate. Another reason for the higher rate of TKIs sensitive *EGFR* mutations could be the use of the more sensitive method ARMS. *EGFR* mutation is a somatic mutation, the detection of which requires a highly specific and sensitive method, for *EGFR* mutant cells are mixed with wild type cells in the tumor sample. ARMS is based on allele specific polymerase chain reaction with a sensitivity at 1% (this means that at least 1% mutant DNAs can be detected within a “normal” DNA background via ARMS), which also makes the results more accurate.

In a subgroup analysis where the subtypes of *EGFR* mutations were studied, we found that exon 19 deletion and exon 21 L858R point mutation were the two dominant subtypes of TKIs sensitive *EGFR* mutations in Chinese patients with NSCLC, comprising 43.4% and 48.1% of all the TKIs sensitive *EGFR* mutations respectively. It is interesting that the frequency was not consistent with previous Asian population-based studies [13,22,23], which demonstrated that exon 19 in-frame deletion was more frequent. It could be explained by unselected tumor subtypes and stages in our study, ethnic variations between Chinese and other populations, or sampling errors.

Two major TKIs resistant *EGFR* mutations (exon 20 insertion and T790M point mutation) were found in Chinese patients with NSCLC prior to any treatment. Exon 20 insertion mutations are associated with primary TKIs resistance. It can promote the activation of EGFR kinase domain, leading to carcinogenesis. It can also affect ATP and the affinity of EGFRs to gefitinib or erlotinib, causing the resistance against gefitinib or its sister drug. Patients harboring exon 20 insertion mutations should receive irreversible inhibitors rather than gefitinib or erlotinib. T790M point mutation in exon 20 is responsible for approximately 50% patients with acquired resistance against TKIs [24]. This mutation does not reduce the affinity of gefitinib or erlotinib to the receptors but it enhances the affinity to ATP and thereby causes resistance. However, *de novo* T790M mutations were found in Chinese patients with NSCLC before administration of TKIs in our study, indicating that T790M could also lead to primary resistance against TKIs; this might confirm that a low frequency T790M mutation may have been present in the primary cancer, but under the selective pressure imposed by targeted therapies it may expand and lead to TKIs resistance [25]. As reported by Tetsuya Mitsudomi *et al.* ([14]; ASCO 2012, abstract 7521), compared with patients harboring T790M wild type, the T790M mutant counterparts could enjoy longer progress free survival (PFS) and overall survival (OS), no matter what type of treatments they received (TKIs or chemotherapy). Thus, the identification of these resistant *EGFR* mutations is as important as the identification of TKIs sensitive ones for the treatment patterns of NSCLC.

In addition, our study failed to find a significant association between the subtypes of *EGFR* mutations and gender, smoking status, and tumor histology, which was different from the study of Tanaka *et al.* [13], who reported that there was a gender difference in *EGFR* mutation subtypes. Tumor stages (unselected *vs.* advanced stage), mutation detection methods (ARMS *vs.* PNA-LNA PCR clamp), specimen types (all fresh cases *vs.* fresh cases/archival tissue), racial differences (Chinese *vs.* Japanese) or sampling errors are likely to be the underlying causes of the discordance between our study and Tanaka’s. More studies are required to elucidate such discord.

Even though targeting therapy is a brand new strategy for the treatment of non-small cell lung cancer, surgical resection is still regarded as the predominant method of controlling the tumor. Apart from the early stage cases, patients at late stages, provided the risks of the procedure are low, could also undergo surgeries for more sufficient tumor samples for pathologic and molecular diagnoses rather than needle biopsies and benefit the most from treatments. The explanations for this are mentioned above. In addition to that, drug resistance and heterogeneity of the *EGFR* mutations that results from tumor heterogeneity, contribute to the rationale for resections when the disease recurs or metastasizes, for significantly more adequate tissue could make the reevaluations of the pathologic and molecular status of the diseases more accurate.

Using ARMS to detect *EGFR* mutations in NSCLC, led to three limitations in our study. First, the primers in the ADx EGFR Mutations Detection Kit we used are designed for the 29 already known *EGFR* mutations, in some rare instances polymorphisms may be present that would not be recognized by this assay. Second, although ARMS is sensitive, routinely being able to detect at least 1% mutant in a normal DNA background, when the DNA concentration is below that level, the results would be false-negative; also, if the samples are contaminated, the results would be less accurate. Third, compared with DNA sequencing, ARMS is not readily available and less economic, although it is superior to sequencing in both sensitivity and robustness on a large and diverse set of clinical tumor samples.

## 4. Experimental Section

### 4.1. Patients and Specimen Sampling

Fresh tumor specimens were obtained from 697 consecutive Chinese patients with NSCLC who underwent surgical resection at the Department of Thoracic Surgery, Tangdu Hospital (Xi’an, Shaanxi, China) during the period from January 2012 to August 2013, including 312 patients who underwent video assisted thoracoscopic lobectomy and 385 patients who underwent open thoracotomy. All the patients suffered from NSCLC for the first time, and none of them received any treatment (including TKIs) before operation. Clinical data of each patient were retrieved from their medical records. Prior to the study, written informed consent was obtained from all patients for the use of their tumor samples for molecular and pathologic analysis. The study protocol was approved by the ethics committee of Tangdu Hospital and performed according to principles of good clinical practice.

After surgery, each sample was divided into two parts for pathological re-evaluation and *EGFR* mutation analysis. Pathologic assessment of all samples was performed by two experienced pathologists. All tumor samples were routinely assessed by sectioning, hematoxylin-eosin staining, and visualization under a microscope. Pathological diagnosis was made according to the criteria of the WHO/IASLC Histological Classification of Lung and Pleural Tumors [26]. Patients diagnosed with squamous-cell carcinoma, adenocarcinoma, adenosquamous carcinoma, large-cell carcinoma, sarcomatoid carcinoma, or mucoepidermoid carcinoma were enrolled for further analysis.

### 4.2. *EGFR* Mutation Analysis Using ARMS

Genomic DNA was extracted from fresh tumor specimens using TIANamp Genomic DNA Kit (Tiangen Biotech (Beijing) Co., Ltd, Beijing, China) according to the manufacturer’s instructions. After that, EGFR mutation analysis was performed via ARMS, according to the protocol of the ADx EGFR Mutations Detection Kit (Amoy Diagnostics, Xiamen, China) using the principle of Amplified Refractory Mutation System (ARMS) and covering 29 *EGFR* mutation hotspots from exon 18 to 21. The assay was carried out according to the manufacturer’s protocol with the MX3005P (Stratagene, La Jolla, CA, USA) real-time PCR system. A positive or negative result could be reached if it met the criterion that was defined by the manufacturer’s instructions.

### 4.3. Statistical Analysis

The relationship between *EGFR* mutations and clinical features was analyzed using the Chi-square and Fisher’s Exact test when appropriate (in statistical analysis of R × C contingency table, if more than 20% of cells have expected count less than 5, the Fisher’s exact test is appropriate). Statistical analysis was carried out via SPSS software, version 13.0 (SPSS Company, Chicago, IL, USA, 2004). Two-sided *p* values less than 0.05 were considered to indicate statistical significance.

## 5. Conclusions

In conclusion, for the relatively large sample capacity, more effective tumor samples and the use of a more sensitive method in our study, our data should better represent the current situation of TKIs sensitive *EGFR* mutations in Chinese patients with NSCLS, especially the mutation status of squamous-cell carcinoma, adenocarcinoma and adenosquamous carcinoma. It is recommended that *EGFR* mutation analysis of NSCLC including adenocarcinoma and nonadenocarcinoma should be a routine molecular test in China after lung resection.

## Figures and Tables

**Table 1.** Patient characteristics.

Characteristics	*N*	Mutation *,%	*p*-value
Age			0.066
<60 years	378	116, 30.7%	
≥60 years	319	119, 37.3%	
Gender			<0.001
Male	476	108, 22.7%	
Female	221	127, 57.5%	
Smoking status			0.047
Never	331	124, 37.5%	
Ever	366	111, 30.3%	
Histology			<0.001
Squamous-cell carcinoma	282	41, 14.5%	
Adenocarcinoma **	293	155, 52.9%	
Adenosquamous carcinoma	86	34, 39.5%	
Large-cell carcinoma	11	1, 9.1%	
Sarcomatoid carcinoma	18	2, 11.1%	
Mucoepidermoid carcinoma	7	2, 28.6%	
Stage			0.898
Ia	105	34, 32.4%	
Ib	110	40, 36.4%	
IIa	153	49, 32.0%	
IIb	159	56, 35.2%	
IIIa	155	53, 34.2%	
IIIb	12	2, 16.7%	
IV	3	1, 33.3%	

* Tyrosine Kinase Inhibitors (TKIs) sensitive *EGFR* mutations (19-del, L858R, L861Q, G719X);

** Partition of Chi-square showed higher mutation rate in adenocarcinomas (α′ = 0.003).

**Table 2.** Subtypes of TKIs sensitive *EGFR* mutations according to gender.

Mutation spot	Gender		*p*-value

	Male	Female	
19-Del	48	54	0.864
L858R	51	62	
L861Q	1	3	
G719X	8	8	

19-Del: exon 19 deletion mutation; L858R: exon 21 L858R point mutation; L861Q: exon 21 L861R point mutation; G719X: exon 18 G719X point mutation.

**Table 3.** Subtypes of TKIs sensitive EGFR mutations according to the smoking status.

Mutation spot	Smoking Status		*p*-value

	Ever	Never	
19-Del	22	80	0.202
L858R	34	79	
L861Q	1	3	
G719X	7	9	

19-Del: exon 19 deletion mutation; L858R: exon 21 L858R point mutation; L861Q: exon 21 L861R point mutation; G719X: exon 18 G719X point mutation.

**Table 4.** Subtypes of TKIs sensitive *EGFR* mutation according to histology.

Mutation spot	Pathology				

	Squamous-cell carcinoma	Adeno-carcinoma	Adeno-squamous carcinoma	Others *	*p*-value
19-Del	17	66	17	2	0.671
L858R	19	76	16	2	
L861Q	0	4	0	0	
G719X	5	9	1	1	

19-Del: exon 19 deletion mutation; L858R: exon 21 L858R point mutation; L861Q: exon 21 L861R point mutation; G719X: exon 18 G719X point mutation;

* Others includes large-cell carcinoma, sarcomatoid carcinoma and mucoepideroid carcinoma.

**Table 5.** Listing of features of patients whose tumors harbored EGFR TKI resistant mutations.

Subtype	Age (years)	Gender	Smoking status	Tumor Histology	Stage
T790M	53	Male	Ever	Squamous-cell carcinoma	IIIa
T790M	67	Male	Ever	Adenocarcinoma	IIIb
T790M	65	Male	Never	Squamous-cell carcinoma	Ib
T790M	50	Female	Never	Squamous-cell carcinoma	IIa
T790M	68	Female	Never	Adenocarcinoma	IIb
T790M	71	Female	Never	Adenocarcinoma	Ib
20-Ins	73	Female	Never	Adenocarcinoma	IIIa
20-Ins	62	Female	Never	Adenocarcinoma	IIb

T790M: exon 20 T790M point mutation; 20-Ins: exon 20 insertion mutation.
